# The effect of surgery plus chemoradiotherapy on survival of elderly patients with stage Ⅱ–Ⅲ esophageal cancer: a SEER‐based demographic analysis

**DOI:** 10.1002/cam4.4352

**Published:** 2021-11-19

**Authors:** Hao Lv, Ce Chao, Bin Wang, Zhigang Wang, Yongxiang Qian, Xiaoying Zhang

**Affiliations:** ^1^ Department of Cardiothoracic Surgery The Third Affiliated Hospital of Soochow University Changzhou China

**Keywords:** chemoradiotherapy, elderly patients, esophageal cancer, surgery, survival

## Abstract

**Background:**

The optimal treatment for elderly patients with esophageal cancer (EC) remains controversial. In the present study, we aimed to investigate whether elderly patients with stage II–III EC could benefit from trimodal therapy.

**Methods:**

The selected elderly patients with stage II–III EC between 2004 and 2015 were included in a retrospective cohort study from the Surveillance, Epidemiology, and End Results database. The patients were divided into two groups based on whether or not they underwent surgery. The inverse probability of treatment weighting (IPTW) analysis was used to balance the confounding factors between the two groups. The Cox regression analysis, the log‐rank test, and the Kaplan–Meier curves were conducted to identify the survival benefits of different treatment regimes.

**Results:**

A total of 1596 patients were included in this cohort study, in which 278 patients underwent surgery. In the combination of chemoradiotherapy and surgery group, there were more male patients, more patients aged between 75 and 79 years, and more married patients in the surgery group. Moreover, there were more patients with adenomatous carcinoma, more patients with a tumor size of less than 5 cm, and more patients with a T3 stage in the combination group. In the survival analysis, patients in the combination group had a longer overall survival (OS) and EC‐specific survival (ECSS). After IPTW analysis, the survival analysis generated similar results. The competitive risk model found that our results were stable. There was still a significant difference in OS and ECSS between the combination group and chemoradiotherapy alone group for esophageal adenocarcinoma (*p* < 0.001).

**Conclusions:**

Elderly patients with stage II–III EC, especially those with adenocarcinoma, could benefit from the combination of surgery and chemoradiotherapy.

## Graphical abstract

The old patients were not off limits to radical and comprehensive treatment. Elderly patients with stage II–III EC, especially those with adenocarcinoma, could benefit from the combination of surgery and chemoradiotherapy.

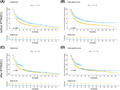

## 1 INTRODUCTION

As a common tumor in digestive system, the annual incidence of esophageal cancer has ranked seventh among all new cases worldwide, and at the meantime the annual mortality has already ranked sixth.^1^ What is more, the aging of population has also led to a continuous increase in the proportion of elderly patients with esophageal cancer. According to a recent survey in China, the risk of suffering from esophageal cancer reached the peak in the age group of 75–84 years old both in male and female patients.^2^ Unfortunately, due to the vulnerable vigilance of elderly population and the lack of typical symptoms during the early stage, the vast majority of elderly patients with esophageal cancer were already in the advanced situation when they were first diagnosed.

Previous studies have confirmed that since the invasive depth of esophageal cancer exceeded the submucosa (T1b), there might be a high likelihood of lymph node metastasis, even a nodal skip metastasis, which was caused by the complex lymphatic drainage networks in the esophageal wall.^3^, ^4^ Thus, as the guideline recommended, the reasonable treatment for patients with stage Ⅱ–Ⅲ esophageal cancer is the combination of surgery and chemoradiotherapy (SCRT), especially with the preoperative neoadjuvant chemoradiotherapy.^5^ However, several retrospective studies have demonstrated that the deteriorative physical condition and the multiple comorbidities of elderly patients caused a significant increase in terms of postoperative complications and perioperative mortality.^6^, ^7^, ^8^ Considering these potential adverse outcomes, elderly patients with locally advanced esophageal cancer preferred to receive definitive chemoradiotherapy (CRT) rather than the trimodality therapy including surgery.^9^ To date, whether elderly patients with stage Ⅱ–Ⅲ esophageal cancer could benefit from the trimodality therapy remains controversial.

In this study, we retrieved data from the Surveillance, Epidemiology, and End Results (SEER) database and aimed to compare the short‐ and long‐term survival outcomes between SCRT and CRT for elderly patients with stage Ⅱ–Ⅲ esophageal cancer.

## 2 MATERIALS AND METHODS

### 2.1 Patient population

**FIGURE 1:**
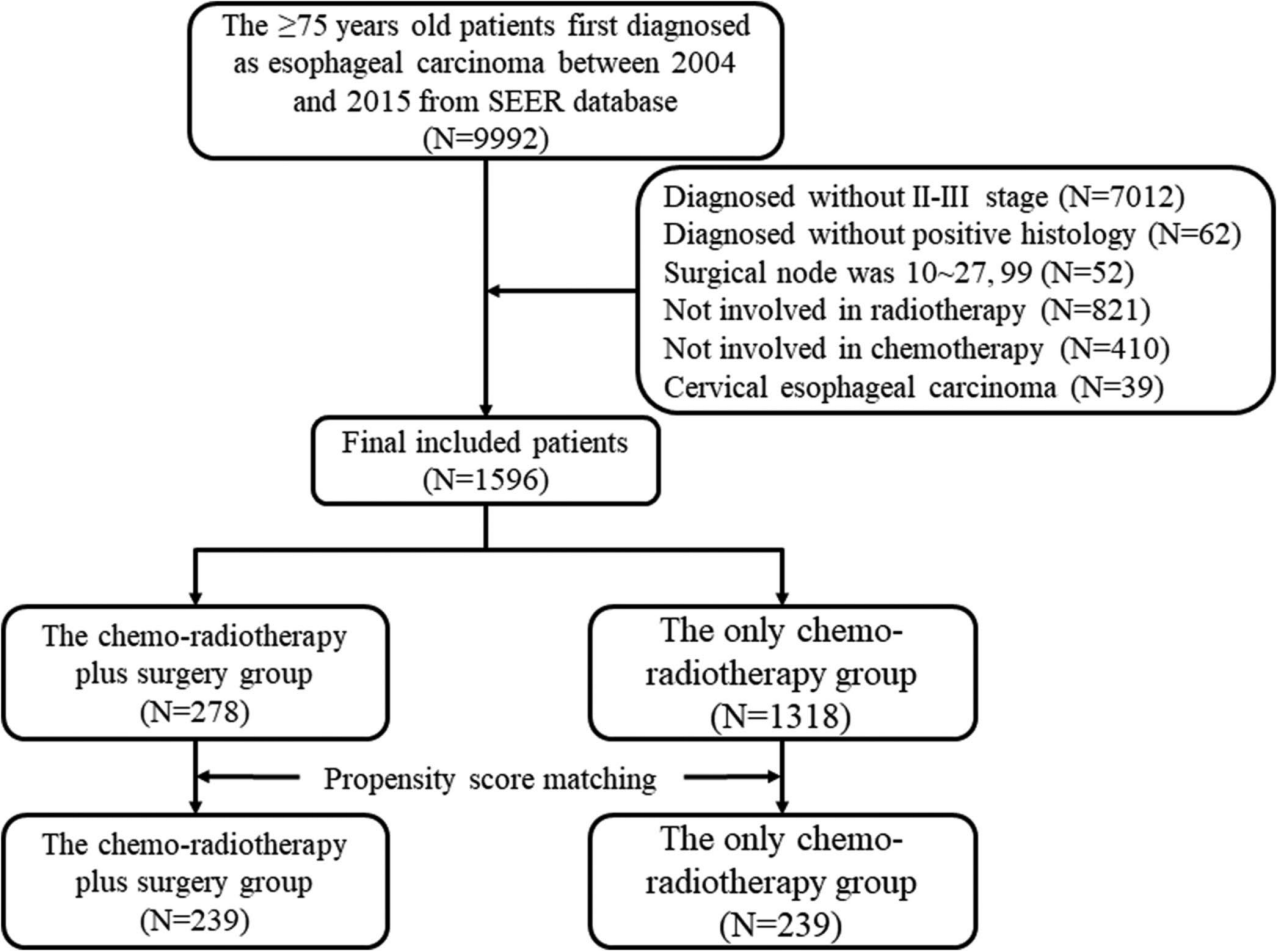
Flowchart of screening out elderly patients with esophageal cancer in this study

Through the National Cancer Institute SEER*Stat software version 8.3.6 (seer.cancer.gov/seerstat), we obtained the clinical data of over 75‐year‐old patients with the first primary esophageal cancer from an incidence‐SEER 18 population‐based registries. A total of 9992 over 75‐year‐old patients who were first primarily diagnosed with malignant tumors of esophagus between 2004 and 2015 were obtained. From these patients, we screened out patients who were diagnosed with stages Ⅱ–Ⅲ based on the sixth edition of TNM staging of esophageal cancer. In addition, patients who did not receive radiation therapy and chemotherapy were excluded. All patients were diagnosed with esophageal cancer according to a positive histology. We divided the patients into surgery group (code 30–90) and non‐surgery group (code 0). At last, eligible 1596 stages Ⅱ–Ⅲ over 75‐year‐old patients were included in this study. The whole flowchart is shown in Figure 1. Patient identification, age of diagnosis, race/ethnicity, year of diagnosis, sex, marital status, primary site, TNM stage, T stage, N stage, histology type, nuclear grade, surgery, chemotherapy, radiation therapy, tumor size, cause‐specific death classification, vital status, and survival month were collected form the SEER database. The overall survival (OS) and esophageal cancer‐specific survival (ECSS) were considered to be the main endpoint of this study. Meanwhile, eligible 368 stages Ⅱ–Ⅲ over 75‐year‐old esophageal cancer patients were obtained and selected from SEER Research Plus Data under the same condition.

### 2.2 Statistical analysis

A Pearson's chi‐squared and Fisher's test were, respectively, used to compare the baseline data and clinical characteristics of patients between the surgery and non‐surgery groups according to the data types and structures. For further analysis, patient age was classified in 5‐year age ranges. And, year of diagnosis was evenly divided into two phases and tumor size was divided by 5 cm. However, everything else remained the same. Univariate and multivariate Cox regression analyses were performed to evaluate independent prognostic factors for OS and ECSS. The survival curves were drawn through the Kaplan–Meier method. To balance the clinicopathological characteristics between two groups, the inverse probability of treatment weighting (IPTW) was conducted using the “MatchIt” R package. We calculated the propensity scores using propensity scores including the following variables: patient age, race, sex, marital status, year of diagnosis, primary site, nuclear grade, histology type, TNM stage, T stage, N stage, and tumor size. Moreover, we used competitive risk model to assess the consistency of the results. All statistical analyses were performed using R software (version 3.6.3) with two‐sided testing and *p* < 0.05 was considered significant.

## 3 RESULTS

### 3.1 Patients characteristics

A total of 1596 patients were included in this cohort study based on exclusion and inclusion principles (Figure 1). Table 1 shows the comparison of the clinicopathological characteristics of 278 patients who underwent surgery and 1318 patients who did not undergo surgery. Compared with the non‐surgery group, more individuals who underwent surgery were White, male, 75–79 years old, or married. In addition, patients with adenomatous carcinoma, patients with tumor size less than 5 cm, and patients with a T3 stage had access to receiving surgery. After IPTW, the baseline characteristics of two groups majority were kept balance (SMD <0.1). Other pathological characteristics, such as grade, histology, tumor size, T stage, and N stage, were hardly balanced between two groups (Table 1).

**TABLE 1:** The clinicopathologic baselines of patients between two groups in esophageal carcinoma patients before propensity score matching

Characteristic	Unweighted population		SMD	Population after IPTW		SMD
	Non‐surgery group (N = 1318)	Surgery group (N = 278)		Non‐surgery group	Surgery group	
Race			0.204			0.058
White	1172 (88.9%)	261 (93.9%)		89.8%	90.7%	
Black	75 (5.7%)	6 (2.2%)		5.1%	4.7%	
Other	69 (5.2%)	11 (4.0%)		5.0%	4.6%	
Unknown	2 (0.1%)	0 (0.0%)		0.1%	0.0%	
Gender			0.284			0.083
Female	384 (29.1%)	48 (17.3%)		27.1%	23.5%	
Male	934 (70.9%)	230 (82.7%)		72.9%	76.5%	
Age group			0.736			0.072
75–79 years	681 (51.7%)	224 (80.6%)		56.7%	53.8%	
80–84 years	447 (33.9%)	53 (19.1%)		31.4%	34.8%	
85+ years	190 (14.4%)	1 (0.4%)		12.0%	11.5%	
Year of diagnosis			0.033			0.042
2004–2009	567 (43.0%)	115 (41.4%)		42.5%	44.6%	
2010–2015	751 (57.0%)	163 (58.6%)		57.5%	55.4%	
Marital status			0.310			0.163
Married	746 (56.6%)	198 (71.2%)		59.2%	66.1%	
Other	519 (39.4%)	74 (26.6%)		37.2%	32.0%	
Unknown	53 (4.0%)	6 (2.2%)		3.7%	1.9%	
Grade			0.299			0.161
I	49 (3.7%)	6 (2.2%)		3.5%	2.7%	
II	479 (36.3%)	91 (32.7%)		35.8%	37.9%	
III	507 (38.5%)	139 (50.0%)		40.4%	34.6%	
IV	20 (1.5%)	8 (2.9%)		1.6%	1.1%	
Unknown	263 (20.0%)	34 (12.2%)		18.7%	23.7%	
Histology			0.642			0.316
EAC	710 (53.9%)	222 (79.9%)		58.4%	51.7%	
ESCC	523 (39.7%)	36 (12.9%)		35.1%	31.7%	
Other	85 (6.4%)	20 (7.2%)		6.6%	16.5%	
Tumor size			0.322			0.214
<5 cm	438 (33.2%)	126 (45.3%)		35.2%	45.6%	
≥5 cm	396 (30.0%)	88 (31.7%)		30.4%	25.0	
Unknown	484 (36.7%)	64 (23.0%)		34.4%	29.3%	
T stage			0.295			0.126
T1	110 (8.3%)	10 (3.6%)		7.5%	6.5%	
T2	304 (23.1%)	60 (21.6%)		22.7%	27.9%	
T3	732 (55.5%)	186 (66.9%)		57.6%	53.1%	
T4	172 (13.1%)	22 (7.9%)		12.2%	12.4%	
N stage			0.120			0.108
N0	531 (40.3%)	100 (36.0%)		39.7%	43.8%	
N1	783 (59.4%)	178 (64.0%)		60.1%	56.2%	
NX	4 (0.3%)	0 (0.0%)		0.3%	0.0%	

Abbreviations: EAC, esophageal adenocarcinoma; ESCC, esophageal squamous cell carcinoma; IPTW, inverse probability treatment weighting; SMD, standardized mean difference.

### 3.2 Identification of independent prognostic factors

Univariate and multivariate Cox regression analyses were performed to identify independent prognostic factors for OS and ECSS in over 75‐year‐old patients with esophageal cancer. In univariate and multivariate analyses for OS, T stage was significantly associated with OS in elderly esophageal cancer patients. Meanwhile, marital status, age group, year of diagnosis, tumor size, and surgery were independent factors affecting the OS of elderly esophageal cancer patients (Table 2). In addition, nuclear grade and N stage instead of tumor size may also be the independent factor in the univariate and multivariate analyses for ECSS (Table 2). After IPTW, sex, race, marital status, age group, year of diagnosis, tumor size, T stage, and surgery all were independent factors affecting the OS and ECSS of elderly esophageal cancer patients. Tumor size and nuclear grade may be the independent factors, respectively, affecting the OS and ECSS of elderly esophageal cancer patients (Table 3).

**TABLE 2:** Univariate and multivariate Cox proportional Hazard analyses for the overall survival and cancer‐specific survival in esophageal carcinoma patients before inverse probability treatment weighting

Characteristic	Overall survival				Esophageal cancer‐specific survival			
	Univariate analysis		Multivariate analysis		Univariate analysis		Multivariate analysis	
	Hazard ratio (95% confidence interval)	*p* value	Hazard ratio (95% confidence interval)	*p* value	Hazard ratio (95% confidence interval)	*p* value	Hazard ratio (95% confidence interval)	*p* value
Surgery								
No	Reference		Reference		Reference		Reference	
Yes	0.63 [0.55, 0.74]	**<0.001**	0.69 [0.59, 0.80]	**<0.001**	0.60 [0.51, 0.72]	**<0.001**	0.63 [0.52, 0.75]	**<0.001**
Marital status								
Married	Reference		Reference		Reference		Reference	
Others	1.17 [1.04, 1.31]	**0.007**	1.12 [1.00, 1.26]	**0.044**	1.20 [1.06, 1.36]	**0.004**	1.16 [1.02, 1.31]	**0.023**
Unknown	1.12 [0.84, 1.49]	0.455	1.06 [0.79, 1.41]	0.706	0.95 [0.67, 1.33]	0.759	0.92 [0.65, 1.30]	0.631
Age group								
75–79 years	Reference		Reference		Reference		Reference	
80–84 years	1.12 [0.99, 1.26]	0.064	1.11 [0.98, 1.25]	0.104	1.11 [0.97, 1.27]	0.123	1.11 [0.97, 1.27]	0.135
85+ years	1.38 [1.16, 1.64]	**<0.001**	1.34 [1.12, 1.60]	**0.001**	1.41 [1.17, 1.70]	**<0.001**	1.38 [1.14, 1.67]	**0.001**
Race								
Black	Reference				Reference			
White	0.97 [0.76, 1.23]	0.796			1.06 [0.80, 1.40]	0.669		
Other	0.82 [0.58, 1.16]	0.259			0.96 [0.66, 1.40]	0.829		
Unknown	0.74 [0.10, 5.36]	0.769			0.00 [0.00, Inf]	0.984		
Gender								
Female	Reference				Reference			
Male	1.10 [0.97, 1.24]	0.136			1.07 [0.94, 1.23]	0.298		
Year of diagnosis								
2004–2009	Reference		Reference		Reference		Reference	
2010–2015	0.82 [0.74, 0.92]	**<0.001**	0.83 [0.74, 0.93]	**0.001**	0.79 [0.70, 0.89]	**<0.001**	0.81 [0.72, 0.92]	**0.001**
Histology								
EAC	Reference				Reference			
ESCC	0.96 [0.86, 1.08]	0.519			0.95 [0.83, 1.08]	0.397		
Others	0.87 [0.70, 1.09]	0.240			0.87 [0.68, 1.12]	0.294		
Tumor size								
<5 cm	Reference		Reference		Reference		Reference	
≥5 cm	1.19 [1.04, 1.36]	**0.012**	1.07 [0.93, 1.22]	0.361	1.23 [1.06, 1.42]	**0.008**	1.07 [0.92, 1.24]	0.397
Unknown	1.24 [1.09, 1.41]	**0.001**	1.16 [1.01, 1.32]	**0.031**	1.23 [1.07, 1.43]	**0.004**	1.14 [0.99, 1.32]	0.077
Grade								
I	Reference				Reference		Reference	
II	1.23 [0.89, 1.69]	0.216			1.37 [0.94, 2.00]	0.102	1.41 [0.99, 2.06]	0.079
III	1.33 [0.96, 1.83]	0.084			1.58 [1.08, 2.29]	**0.018**	1.61 [1.10, 2.35]	**0.014**
IV	1.23 [0.73, 2.05]	0.436			1.29 [0.71, 2.36]	0.399	1.51 [0.83, 2.76]	0.180
Unknown	1.09 [0.78, 1.53]	0.596			1.22 [0.83, 1.81]	0.316	1.19 [0.80, 1.77]	0.380
T stage								
T1	Reference		Reference		Reference		Reference	
T2	0.88 [0.70, 1.10]	0.257	0.94 [0.74, 1.20]	0.642	0.91 [0.70, 1.19]	0.495	1.01 [0.77, 1.34]	0.917
T3	1.18 [0.96, 1.46]	0.117	1.33 [1.07, 1.65]	**0.010**	1.29 [1.01, 1.65]	**0.039**	1.48 [1.15, 1.89]	**0.002**
T4	1.75 [1.37, 2.23]	**<0.001**	1.81 [1.41, 2.33]	**<0.001**	2.00 [1.52, 2.64]	**<0.001**	2.11 [1.59, 2.81]	**<0.001**
N stage								
N0	Reference		Reference		Reference		Reference	
N1	1.11 [0.99, 1.24]	0.074	1.12 [1.00, 1.26]	0.052	1.14 [1.00, 1.28]	**0.044**	1.17 [1.03, 1.33]	**0.018**
NX	2.88 [1.07, 7.70]	**0.035**	1.78 [0.66, 4.83]	0.257	3.38 [1.26, 9.05]	**0.015**	2.02 [0.74, 5.50]	0.171

Statistically significant values are indicated in bold.

Abbreviations: EAC, esophageal adenocarcinomaESCC, esophageal squamous cell carcinoma.

**TABLE 3:** Univariate and multivariate Cox proportional Hazard analyses for the overall survival and cancer‐specific survival in esophageal carcinoma patients after inverse probability treatment weighting

Characteristic	Overall survival				Esophageal cancer‐specific survival			
	Univariate analysis		Multivariate analysis		Univariate analysis		Multivariate analysis	
	Hazard ratio (95% confidence interval)	*p* value	Hazard ratio (95% confidence interval)	*p* value	Hazard ratio (95% confidence interval)	*p* value	Hazard ratio (95% confidence interval)	*p* value
Surgery								
No	Reference		Reference		Reference		Reference	
Yes	0.73 [0.68, 0.79]	**<0.001**	0.79 [0.73, 0.86]	**<0.001**	0.73 [0.67, 0.80]	**<0.001**	0.79 [0.72, 0.86]	**<0.001**
Marital status								
Married	Reference		Reference		Reference		Reference	
Others	1.28 [1.18, 1.39]	**<0.001**	1.27 [1.16, 1.38]	**<0.001**	1.29 [1.18, 1.41]	**<0.001**	1.29 [1.17, 1.42]	**<0.001**
Unknown	1.10 [0.88, 1.38]	0.403	1.07 [0.85, 1.35]	0.539	0.80 [0.60, 1.06]	0.123	0.83 [0.62, 1.11]	0.209
Age group								
75–79 years	Reference		Reference		Reference		Reference	
80–84 years	1.22 [1.12, 1.33]	**<0.001**	1.26 [1.16, 1.38]	**<0.001**	1.25 [1.14, 1.38]	**<0.001**	1.31 [1.19, 1.45]	**<0.001**
85+ years	1.05 [0.93, 1.18]	0.419	1.42 [1.23, 1.64]	**<0.001**	1.17 [1.03, 1.33]	**0.015**	1.64 [1.41, 1.92]	**<0.001**
Race								
Black	Reference		Reference		Reference		Reference	
White	0.69 [0.58, 0.82]	**<0.001**	0.79 [0.66, 0.94]	**0.009**	0.70 [0.58, 0.85]	**<0.001**	0.79 [0.65, 0.96]	**0.020**
Other	0.69 [0.54, 0.87]	**0.002**	0.66 [0.52, 0.84]	**0.001**	0.75 [0.58, 0.98]	**0.034**	0.69 [0.53, 0.91]	**0.007**
Unknown	0.67 [0.09, 4.76]	0.686	0.69 [0.10, 4.92]	0.708	0.00 [0.00, Inf]	0.686	0.00 [0.00, Inf]	0.978
Gender								
Female	Reference		Reference		Reference		Reference	
Male	1.11 [1.01, 1.22]	**0.025**	1.26 [1.14, 1.39]	**<0.001**	1.17 [1.06, 1.30]	**0.002**	1.38 [1.23, 1.54]	**<0.001**
Year of diagnosis								
2004–2009	Reference		Reference		Reference		Reference	
2010–2015	0.86 [0.79, 0.93]	**<0.001**	0.83 [0.76, 0.90]	**<0.001**	0.82 [0.75, 0.89]	**<0.001**	0.81 [0.74, 0.89]	**<0.001**
Histology								
EAC	Reference		Reference		Reference		Reference	
ESCC	1.08 [0.99, 1.17]	0.090	1.09 [1.00, 1.20]	0.063	1.14 [1.04, 1.25]	**0.006**	1.17 [1.06, 1.30]	**0.002**
Others	0.83 [0.74, 0.94]	**0.003**	0.96 [0.83, 1.11]	0.607	0.93 [0.82, 1.05]	0.247	1.11 [0.94, 1.30]	0.225
Tumor size								
<5 cm	Reference		Reference		Reference			
≥5 cm	1.12 [1.02, 1.24]	**0.017**	0.98 [0.89, 1.09]	0.730	1.10 [0.99, 1.22]	0.083		
Unknown	1.15 [1.05, 1.26]	**0.002**	1.11 [1.01, 1.22]	**0.030**	1.10 [1.00, 1.22]	0.054		
Grade								
I	Reference				Reference		Reference	
II	1.16 [0.92, 1.47]	0.211			1.34 [1.02, 1.77]	**0.038**	1.33 [1.00, 1.75]	**0.048**
III	1.09 [0.86, 1.38]	0.490			1.30 [0.98, 1.71]	0.064	1.30 [0.98, 1.72]	0.066
IV	1.11 [0.74, 1.65]	0.614			1.15 [0.71, 1.84]	0.573	1.34 [0.83, 2.16]	0.228
Unknown	0.94 [0.74, 1.20]	0.627			1.11 [0.83, 1.47]	0.482	1.10 [0.82, 1.47]	0.528
T stage								
T1	Reference		Reference		Reference		Reference	
T2	0.91 [0.77, 1.07]	0.242	1.04 [0.87, 1.25]	0.636	0.89 [0.74, 1.07]	0.226	1.05 [0.86, 1.29]	0.614
T3	1.16 [0.99, 1.35]	0.065	1.41 [1.20, 1.66]	**<0.001**	1.17 [0.98, 1.39]	0.083	1.48 [1.24, 1.78]	**<0.001**
T4	2.37 [1.98, 2.84]	**<0.001**	2.60 [2.17, 3.13]	**<0.001**	2.62 [2.15, 3.18]	**<0.001**	2.97 [2.43, 3.63]	**<0.001**
N stage								
N0	Reference		Reference		Reference		Reference	
N1	1.26 [1.16, 1.36]	**<0.001**	1.22 [1.12, 1.33]	**<0.001**	1.27 [1.16, 1.38]	**<0.001**	1.26 [1.15, 1.39]	**<0.001**
NX	3.58 [1.34, 9.57]	**0.011**	1.88 [0.70, 5.08]	0.212	4.16 [1.56, 11.12]	**0.004**	2.23 [0.82, 6.04]	0.115

Statistically significant values are indicated in bold.

Abbreviations: EAC, esophageal adenocarcinoma; ESCC, esophageal squamous cell carcinoma.

### 3.3 Survival analysis for OS and ECSS

**FIGURE 2:**
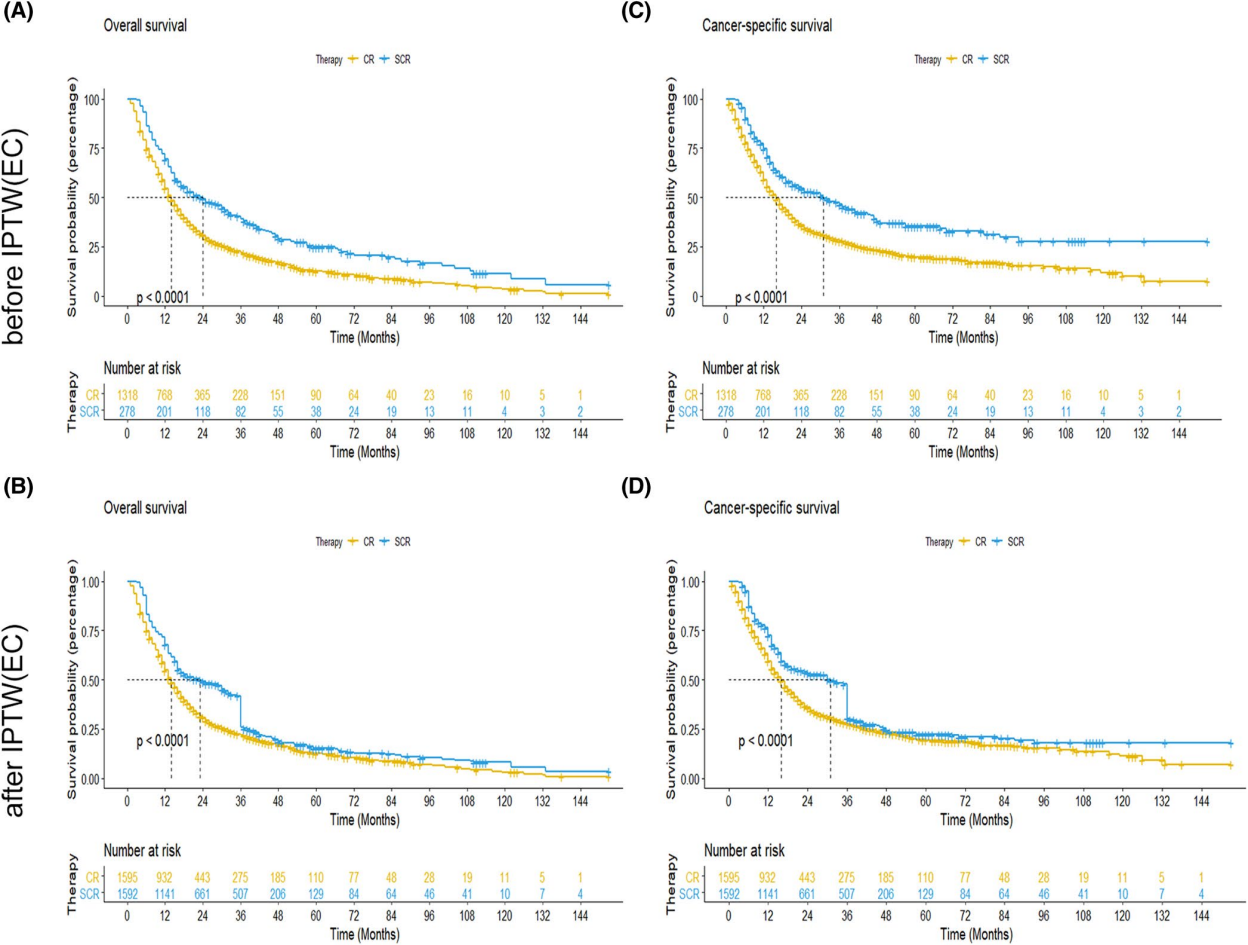
Kaplan–Meier curves for OS and ECSS of patients with esophageal cancer. (A and B): Kaplan–Meier OS curves between chemoradiotherapy plus surgery group and only chemoradiotherapy group before (A) and after (B) inverse probability of treatment weighting, (C and D): Kaplan–Meier ECSS curves between chemoradiotherapy plus surgery group and only chemoradiotherapy group before (C) and after (D) inverse probability of treatment weighting. Abbreviations: ECSS, esophageal cancer‐special survival; OS, overall survival; CR, chemoradiotherapy group; SCR, surgery plus chemoradiotherapy group

The Kaplan–Meier analysis indicated that patients with chemoradiotherapy plus surgery had longer OS and ECSS compared with the ones with only chemoradiotherapy (Figure 2A,B). The 1‐, 3‐, and 5‐year OS rates were 69.42%, 39.02%, and 24.71% in the chemoradiotherapy plus surgery group and the 1‐, 3‐, and 5‐year OS rates were 54.58%, 21.85%, and 12.44% in the only chemoradiotherapy group, respectively. Likewise, the 1‐, 3‐, and 5‐year ECSS rates of the chemoradiotherapy plus surgery group were higher than the ones with only chemoradiotherapy group (1‐year ECSS rate: 74.59% vs. 59.08%, 3‐year ECSS rate: 46.31% vs. 27.99%, and 5‐year ECSS rate: 35.34% vs. 19.64%). After IPTW, the clinicopathologic baselines of two group majorly achieved balance (Table 1). The OS and ECSS survival curve after IPTW showed the similar results with before IPTW in chemoradiotherapy plus surgery group and only chemoradiotherapy group (Figure 2C,D). In addition, the similar results were obtained using stages Ⅱ–Ⅲ over 75‐year‐old esophageal cancer patients between 2016 and 2017 from SEER Research Plus Data. The chemoradiotherapy plus surgery group was associated with better OS and ECSS of stages Ⅱ–Ⅲ over 75‐year‐old esophageal cancer patients (Figure S1).

### 3.4 Subgroup analysis

**FIGURE 3:**
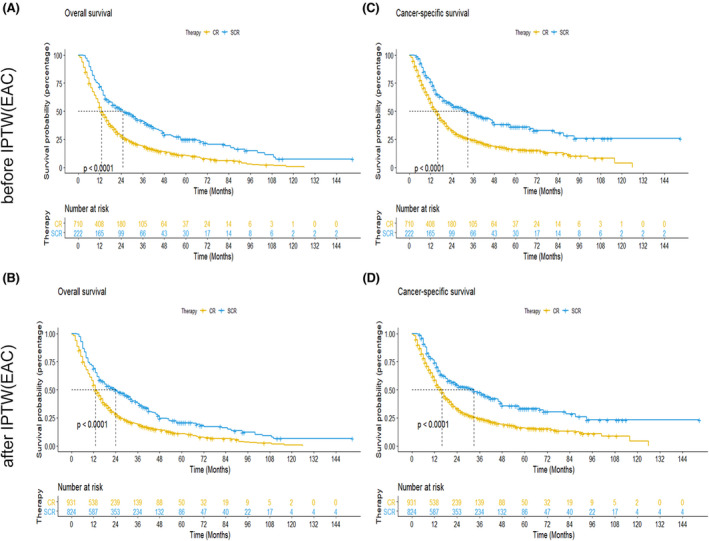
Kaplan–Meier curves for OS and ECSS of patients with esophageal adenocarcinoma. (A and B): Kaplan–Meier OS curves between chemoradiotherapy plus surgery group and only chemoradiotherapy group before (A) and after (B) inverse probability of treatment weighting, (C and D): Kaplan–Meier ECSS curves between chemoradiotherapy plus surgery group and only chemoradiotherapy group before (C) and after (D) inverse probability of treatment weighting. Abbreviations: ECSS, esophageal cancer‐special survival; OS, overall survival; CR, chemoradiotherapy group; SCR, surgery plus chemoradiotherapy group

**FIGURE 4:**
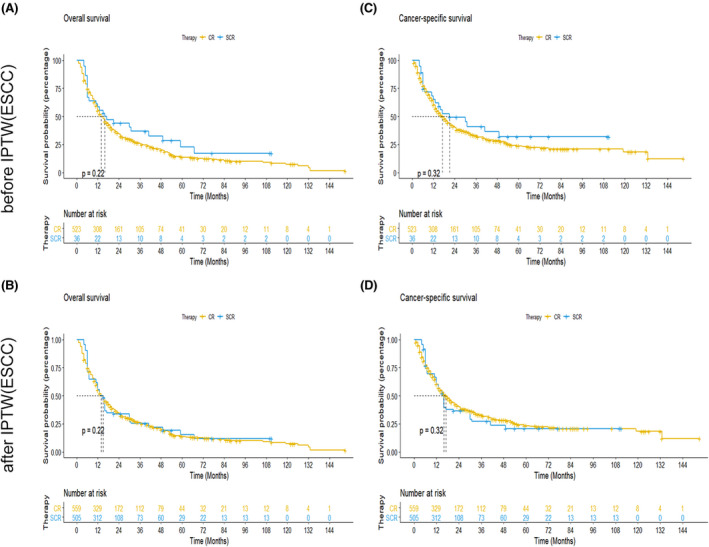
Kaplan–Meier curves for OS and ECSS of patients with esophageal squamous cell carcinoma. (a and b): Kaplan–Meier OS curves between chemoradiotherapy plus surgery group and only chemoradiotherapy group before (A) and after (B) inverse probability of treatment weighting, (C and D): Kaplan–Meier ECSS curves between chemoradiotherapy plus surgery group and only chemoradiotherapy group before (C) and after (D) inverse probability of treatment weighting. Abbreviations: ECSS, esophageal cancer‐special survival; OS, overall survival; CR, chemoradiotherapy group; SCR, surgery plus chemoradiotherapy group

Considering the variation in the treatment of esophageal cancer among different pathological types, we conducted the Kaplan–Meier analysis between chemoradiotherapy plus surgery group and only chemoradiotherapy group in esophageal squamous cell carcinoma (ESCC) and esophageal adenocarcinoma (EAC). In EAC, patients with chemoradiotherapy plus surgery had longer OS and ECSS compared with the ones with only chemoradiotherapy before and after IPTW (Figure 3A–D). While, the survival advantage of surgery was not significant in ESCC before IPTW (Figure 4A,B). Similarly, the survival benefit of OS and ECSS was not significant between chemoradiotherapy plus surgery group and only chemoradiotherapy group after PSM (Figure 4C,D).

### 3.5 Competitive risk model

We performed competitive risk model to assess the stability of results. The cumulative incidence curve indicated that the cancer‐special death between chemoradiotherapy plus surgery group and only chemoradiotherapy group was significant for EAC but ESCC (Figure S2A–C). Univariate and multivariate competitive risk model confirmed that marital status, age group, year of diagnosis, nuclear grade, T stage, N stage, and surgery were independent factors affecting patient's survival (Table S1). After IPTW, histology and sex instead of nuclear grade may be independent factors affecting the prognosis of patients (Table S1).

## 4 DISCUSSION

Esophageal cancer has caused a tremendous burden on human health due to its poor prognosis. The overall 5‐year survival rate of esophageal cancer is barely maintained at 10%–30% all over the world.^10^ All along, clinicians are constantly exploring new technologies and methods to improve the long‐term prognosis, such as the application of neoadjuvant chemoradiotherapy, minimally invasive esophagectomy, and the latest immunotherapy or targeted therapy. Accordingly, both the univariate and multivariate cox regression analyses in our study showed that the period of diagnosis was a significant risk factor for survival. Patients diagnosed in latter 6 years had a better OS, which we inferred, was attribute to recent advances in treatment. However, Molena et al. previously evaluated a SEER‐Medicare cohort that contained 5072 esophageal cancer patients aged over 65 years and found that 34.74% of these patients received no clinical treatment, the majority of them (48.49%) received definitive chemoradiation.^11^ Similarly, this phenomenon was also existed in our study with only 278 patients included in SCRT group while 1318 patients included in CRT group. In addition, nearly 81% of the patients in SCRT group were aged 75–79 years. Interestingly, in this study, we found that the combination of surgery and chemoradiotherapy significantly improved the survival outcomes compared to chemoradiotherapy alone for elderly patients with stage Ⅱ–Ⅲ esophageal cancer.

At present, high‐level evidence from randomized controlled trials (RCTs) for elderly patients with esophageal cancer is still lacking. Clinically, patients over 75 years old were generally excluded from RCTs. For instance, the eligible patients recruited in the Chemoradiotherapy for esophageal cancer followed by Surgery Study (CROSS trial) only aged 18–75 years.^12^ A previous systematic review evaluated two RCTs and found that the addition of esophagectomy to chemoradiotherapy failed to improve the OS in locally advanced esophageal cancer, but increased treatment‐related mortality unfortunately.^13^ In addition, a series of single‐center retrospective studies and meta‐analysis showed higher rates of postoperative complications and mortality in the elderly group when compared to the younger patients.^6^, ^14^, ^15^, ^16^ Actually, whether advanced age by itself has a negative effect on surgical outcomes is still under debate. Interestingly, a growing evidence revealed that esophagectomy has played a positive role in the treatment of elderly patients with esophageal cancer. Fang et al. reported no significant difference in overall or cause‐specific 5‐year survival in patients aged 70 and over undergoing esophagectomy with three‐field lymph node dissection.^17^ Based on the Charlson comorbidity index, Paulus et al. compared the octogenarian group with the case‐matched younger group and revealed similar outcomes in terms of postoperative morbidity (56% vs. 45%), mortality (9% vs. 9%), and length of hospital stay (18 days vs. 13 days).^18^ Similarly, a recent meta‐analysis reported that in its subgroup analysis, the 30 days, 90 days, and in‐hospital mortality rates in older patients (≥75 years) undergoing the minimally invasive esophagectomy were comparable to younger counterparts.^19^

It is well known that the different combinations of surgery, chemotherapy, and radiotherapy have become the mainstay of treatment for stage II–III esophageal cancer in recent decades, yet the therapeutic effects remain variable and nonuniform. The OEO2 trial conducted by Allum et al. evaluated the role of neoadjuvant chemotherapy and showed an improved 5‐year survival of 23.0% compared with 17.1% for surgery alone. In addition, this survival benefit was achieved both in esophageal squamous cell carcinoma and adenocarcinoma.^20^ Furthermore, the remarkable CROSS trail was initiated to compare the preoperative chemoradiotherapy plus surgery with surgery alone in patients with resectable esophageal tumors. The trimodality approach presented significant better OS but similar postoperative complications and in‐hospital mortality compared to surgery alone.^12^ On the other hand, Hategan et al. analyzed 102 patients and found that the trimodality therapy offered higher rates of 2‐year survival and 5‐year survival compared with the definitive chemoradiotherapy in their cancer center.^21^ In contrast, data from a recent meta‐analysis only showed a non‐significant trend toward survival benefit for trimodality therapy compared to definitive chemoradiotherapy when considering the equal baseline conditions in different groups.^22^

Previously, both in retrospective and prospective studies, definitive chemoradiotherapy has been confirmed to be well tolerated and effective in elderly patients with esophageal cancer.^23^, ^24^ However, given concern for the additional surgical trauma, the safety and efficacy of trimodality therapy for elderly patients are still uncertain. Interestingly, our study demonstrated better OS and cancer‐specific survival for SCRT over CRT in elderly patients, while attribute to the limited information from SEER database, the precise combination forms, and regimens of treatment were not fully clear. What is more, the further inverse probability treatment weighting analysis revealed the survival advantage in SCRT group as well. A recent retrospective study with 89 elderly patients (≥75 years) showed no cases of 30‐day or 90‐day mortality but favorable OS and progression‐free survival in patients who underwent the trimodality therapy.^25^ A multi‐institutional analysis evaluated 571 patients from three high‐volume cancer centers in USA and found that elderly patients with trimodality therapy experienced more postoperative cardiac and pulmonary toxicities than the younger counterparts.^26^ Nevertheless, authors pointed out that although there was a higher risk of 90‐day mortality (5.4 vs. 2.2%), the disease‐free survival was comparable between the two cohorts, suggested that the aggressive trimodality therapy is a reasonable option for suitable older patients.

We also noticed the different distribution of histological subtypes between the cohorts in our study. Only 12.9% of the patients in SCRT group were squamous cell carcinoma (SCC) cases, while in CRT group the proportion reached 39.7%. Apparently, most of the elderly patients with SCC in our study received chemoradiotherapy alone. We speculated that this tendency was mainly based on the published results that chemoradiotherapy showed better curative effect on SCC.^12^, ^13^, ^27^ Accordingly, we further conducted a subgroup analysis among the different histological subtypes. The Kaplan–Meier analysis revealed that SCRT obtained a significantly better OS among elderly patients with adenocarcinoma (AC) compared to CRT alone. However, consistent with the results reported by Koeter et al,^28^ it seemed that the addition of surgery to chemoradiotherapy did not significantly improve the survival outcome for those with SCC. But a similar critical issue that cannot be ignored is the small sample size of trimodality therapy included in both SCC groups. As we all known, differ from the high incidence of esophageal AC in western countries, the predominant histological subtype in Asia, especially in China, is SCC.^1^ Thus, more large sample data of elderly patients from these regions are urgently needed to elevate the level of relevant evidence.

The advantage of our study is the utilization of SEER database. Based on its huge volume of population in real world, we can directly obtain sufficient clinical information and accurate survival data toward target patients, especially for those with relatively rare characteristics. On the other hand, our study also has several limitations. First, the free version of SEER database cannot provide either the definite regimen and sequence of chemotherapy, or the detailed dose of radiotherapy. It is commonly believed that variable chemoradiotherapy regimen standards were applied in different histological subtypes and age groups. Second, the lack of information on comorbidities and performance status of included elderly patients made it hard to evaluate the extent of selection bias among groups. Patients receiving trimodality therapy were more likely to have better physical conditions before treatment. Third, the staging system adopted in present study was the sixth edition of AJCC, the main difference was that there might be some patients with stage T4b who are now reclassified as stage IV in the latest eighth edition. Fourth, the SEER database majorly collected the American patients. Therefore, there were small part ESCC patients. We hope more associated studies were performed to verify our result for the Asian population. Lastly, we cannot eliminate the possibility that the elderly patients underwent initial preoperative chemoradiation finally did not receive surgery on account of latter physical reserve deterioration, disease progression, or decline of financial capacity.

## 5 CONCLUSION

In summary, our study indicate that elderly patients aged 75 years and over with stage II–III esophageal cancer, especially for EAC patients, can get a survival benefit from the trimodality therapy, though there may be an increased risk of postoperative complications and mortality compared with chemoradiotherapy alone. It reminds us that further research should focus on establishing a reasonable comprehensive evaluation system which can take the performance status, comorbidities, rest life quality, and even financial standing into consideration, in order to identify the appropriate patients in this unique population.

## 6 ETHICS STATEMENT

The data were derived from the SEER database, and it was unnecessary to obtain patient consent again.

## CONFLICT OF INTEREST

No conflict of interest exits in the submission of this manuscript.

## AUTHOR CONTRIBUTIONS

(I) Conception and design: HL, CC, and XY‐Z. (II) Administrative support: XY‐Z.

(III) Provision of study materials or patients: CC. (IV) Collection and assembly of data: HL, CC, and YX‐Q. (V) Data analysis and interpretation: HL, CC, BW, and ZG‐W. (VI) Manuscript writing: HL and CC. (VII) Final approval of manuscript: All authors.

## Supporting information

Fig S1

Fig S2

Table S1

## Data Availability

The datasets used and/or analyzed during the current study are available from the first author upon reasonable request.

## References

[1] Bray F , Ferlay J , Soerjomataram I , Siegel RL , Torre LA , Jemal A . Global cancer statistics 2018: GLOBOCAN estimates of incidence and mortality worldwide for 36 cancers in 185 countries. CA Cancer J Clin. 2018;68:394‐424. 30207593 10.3322/caac.21492

[2] Chen R , Zheng RS , Zhang SW , et al. Analysis of incidence and mortality of esophageal cancer in China, 2015. Zhonghua Yu Fang Yi Xue Za Zhi. 2019;53:1094‐1097. 31683393 10.3760/cma.j.issn.0253-9624.2019.11.004

[3] Molena D , Schlottmann F , Boys JA , et al. Esophagectomy following endoscopic resection of submucosal esophageal cancer: a highly curative procedure even with nodal metastases. J Gastrointest Surg. 2017;21:62‐67. 27561633 10.1007/s11605-016-3210-3

[4] Zhuge L , Wang S , Xie J , et al. A model based on endoscopic morphology of submucosal esophageal squamous cell carcinoma for determining risk of metastasis on lymph nodes. J Thorac Dis. 2018;10:6846‐6853. 30746230 10.21037/jtd.2018.11.77 PMC6344677

[5] Ajani JA , D’Amico TA , Bentrem DJ , et al. Esophageal and esophagogastric junction cancers, version 2.2019, NCCN clinical practice guidelines in oncology. J Natl Compr Canc Netw. 2019;17:855‐883. 31319389 10.6004/jnccn.2019.0033

[6] Cijs TM , Verhoef C , Steyerberg EW , et al. Outcome of esophagectomy for cancer in elderly patients. Ann Thorac Surg. 2010;90:900‐907. 20732515 10.1016/j.athoracsur.2010.05.039

[7] Jing W , Guo H , Kong L , et al. Clinical outcomes of elderly patients (>/=70 years) with resectable esophageal squamous cell carcinoma who underwent esophagectomy or chemoradiotherapy: a retrospective analysis from a single cancer institute. Medicine. 2016;95:e5630. 27977606 10.1097/MD.0000000000005630 PMC5268052

[8] Tapias LF , Muniappan A , Wright CD , et al. Short and long‐term outcomes after esophagectomy for cancer in elderly patients. Ann Thorac Surg. 2013;95:1741‐1748. 23500043 10.1016/j.athoracsur.2013.01.084 PMC3732120

[9] Shao MS , Wong AT , Schwartz D , Weiner JP , Schreiber D . Definitive or preoperative chemoradiation therapy for esophageal cancer: patterns of care and survival outcomes. Ann Thorac Surg. 2016;101:2148‐2154. 27016842 10.1016/j.athoracsur.2015.12.056

[10] Allemani C , Matsuda T , Di Carlo V , et al. Global surveillance of trends in cancer survival 2000–14 (CONCORD‐3): analysis of individual records for 37 513 025 patients diagnosed with one of 18 cancers from 322 population‐based registries in 71 countries. Lancet. 2018;391:1023‐1075. 29395269 10.1016/S0140-6736(17)33326-3 PMC5879496

[11] Molena D , Stem M , Blackford AL , Lidor AO . Esophageal cancer treatment is underutilized among elderly patients in the USA. J Gastrointest Surg. 2017;21:126‐136. 27527093 10.1007/s11605-016-3229-5 PMC5637537

[12] van Hagen P , Hulshof M , van Lanschot J , et al. Preoperative chemoradiotherapy for esophageal or junctional cancer. N Engl J Med. 2012;366:2074‐2084. 22646630 10.1056/NEJMoa1112088

[13] Vellayappan BA , Soon YY , Ku GY , Leong CN , Lu JJ , Tey JC . Chemoradiotherapy versus chemoradiotherapy plus surgery for esophageal cancer. Cochrane Database Syst Rev. 2017;(8):CD010511. 28829911 10.1002/14651858.CD010511.pub2 PMC6483706

[14] Han Y , Liu S , Guo W , Zhang Y , Li H . Clinical outcomes of oesophagectomy in elderly versus relatively younger patients: a meta‐analysis. Interact Cardiovasc Thorac Surg. 2019;29:897‐905. 31765482 10.1093/icvts/ivz208

[15] Klevebro F , Garritano S , Scandavini CM , et al. Surgical outcomes of oesophagectomy or gastrectomy due to cancer for patients >/=75 years of age: a single‐centre cohort study. ANZ J Surg. 2019;89:228‐233. 30151854 10.1111/ans.14761

[16] Kozuki R , Watanabe M , Toihata T , et al. Treatment strategies and outcomes for elderly patients with locally advanced squamous cell carcinoma of the esophagus. Surg Today. 2021. [Online ahead of print]. 10.1007/s00595-021-02348-9 34331129

[17] Fang W , Igaki H , Tachimori Y , Sato H , Daiko H , Kato H . Three‐field lymph node dissection for esophageal cancer in elderly patients over 70 years of age. Ann Thorac Surg. 2001;72:867‐871. 11565672 10.1016/s0003-4975(01)02896-x

[18] Paulus E , Ripat C , Koshenkov V , et al. Esophagectomy for cancer in octogenarians: should we do it? Langenbecks Arch Surg. 2017;402:539‐545. 28303419 10.1007/s00423-017-1573-x

[19] Baranov NS , Slootmans C , van Workum F , Klarenbeek BR , Schoon Y , Rosman C . Outcomes of curative esophageal cancer surgery in elderly: a meta‐analysis. World J Gastrointest Oncol. 2021;13:131‐146. 33643529 10.4251/wjgo.v13.i2.131 PMC7896422

[20] Pasquali S , Yim G , Vohra RS , et al. Survival after neoadjuvant and adjuvant treatments compared to surgery alone for resectable esophageal carcinoma: a network meta‐analysis. Ann Surg. 2017;265:481‐491. 27429017 10.1097/SLA.0000000000001905

[21] Hategan M , Cook N , Prewett S , Hindmarsh A , Qian W , Gilligan D . Trimodality therapy and definitive chemoradiotherapy for esophageal cancer: a single‐center experience and review of the literature. Dis Esophagus. 2015;28:612‐618. 24863560 10.1111/dote.12242

[22] Voeten DM , den Bakker CM , Heineman DJ , Ket JCF , Daams F , van der Peet DL . Definitive chemoradiotherapy versus trimodality therapy for resectable oesopesope: meta‐analyses and systematic review of literature. World J Surg. 2019;43:1271‐1285. 30607604 10.1007/s00268-018-04901-z

[23] Hino K , Kajiwara T , Nishina T , et al. Tolerability of definitive chemoradiotherapy in elderly patients with esophageal cancer. Gan to Kagaku Ryoho. 2020;47:1577‐1581. 33268731

[24] Servagi‐Vernat S , Crehange G , Bonnetain F , Mertens C , Brain E , Bosset JF . Chemoradiation in elderly esophageal cancer patients: rationale and design of a phase I/II multicenter study (OSAGE). BMC Cancer. 2017;17:483. 28705182 10.1186/s12885-017-3465-4 PMC5508772

[25] Rahimy E , Koong A , Toesca D , et al. Outcomes and tolerability of definitive and preoperative chemoradiation in elderly patients with esophageal cancer: a retrospective institutional review. Adv Radiat Oncol. 2020;5:1188‐1196. 33305080 10.1016/j.adro.2020.05.001 PMC7718494

[26] Lester SC , Lin SH , Chuong M , et al. A multi‐institutional analysis of trimodality therapy for esophageal cancer in elderly patients. Int J Radiat Oncol Biol Phys. 2017;98:820‐828. 28476435 10.1016/j.ijrobp.2017.02.021

[27] Chen H , Zhou L , Yang Y , Yang L , Chen L . Clinical effect of radiotherapy combined with chemotherapy for non‐surgical treatment of the esophageal squamous cell carcinoma. Med Sci Monit. 2018;24:4183‐4191. 29915168 10.12659/MSM.910326 PMC6040238

[28] Koeter M , van Putten M , Verhoeven RHA , Lemmens V , Nieuwenhuijzen GAP . Definitive chemoradiation or surgery in elderly patients with potentially curable esophageal cancer in the Netherlands: a nationwide population‐based study on patterns of care and survival. Acta Oncol. 2018;57:1192‐1200. 29528262 10.1080/0284186X.2018.1450521

