# Supplementary figures and images for: The effect of surgery plus chemoradiotherapy on survival of elderly patients with stage Ⅱ–Ⅲ esophageal cancer: a SEER‐based demographic analysis
Source: Cancer Med. 2021 Nov 19;10(23):8483–96. doi: 10.1002/cam4.4352 (PMC8633220; doi:10.1002/cam4.4352)

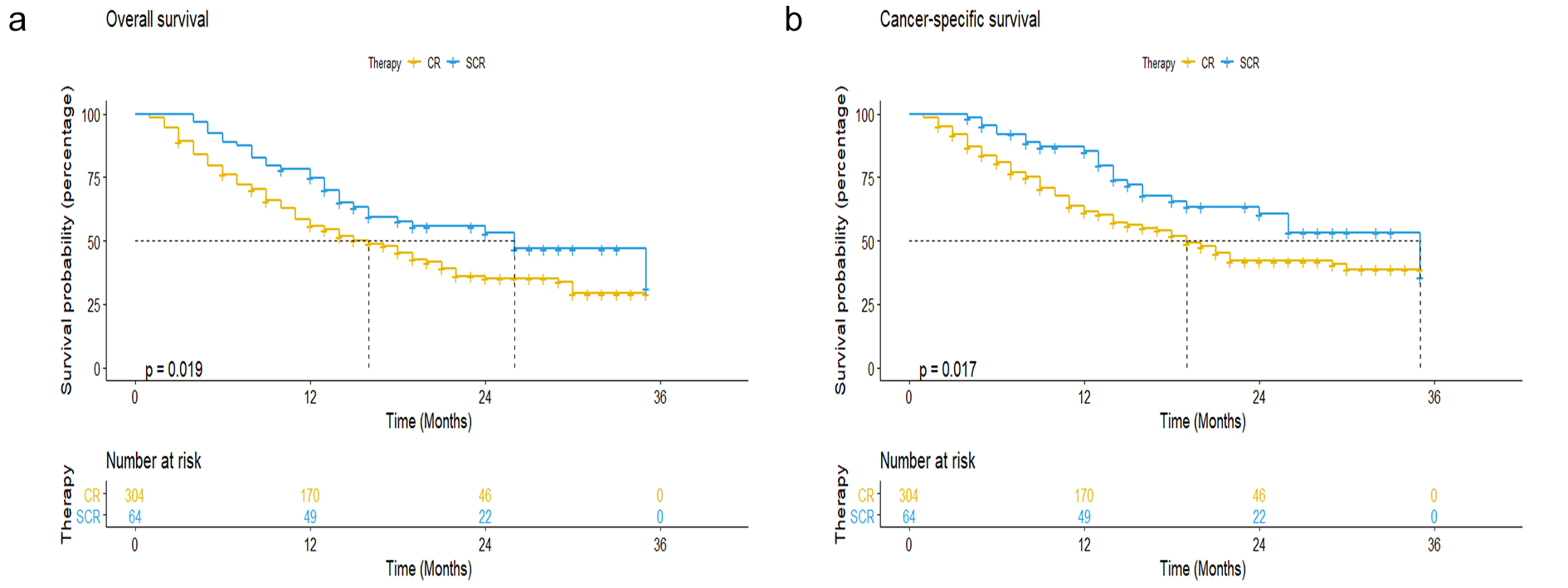

Supplement: Supplementary file 1 — Fig S1 [file CAM4-10-8483-s002.tif]

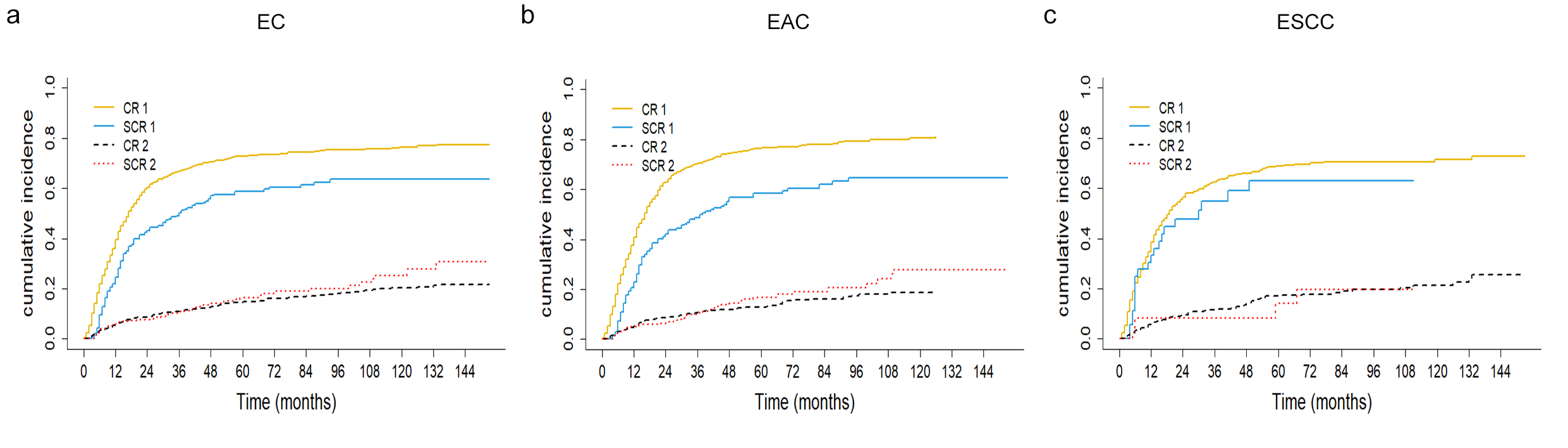

Supplement: Supplementary file 2 — Fig S2 [file CAM4-10-8483-s003.tif]
