# Supplementary material for: The effect of surgery plus chemoradiotherapy on survival of elderly patients with stage Ⅱ–Ⅲ esophageal cancer: a SEER‐based demographic analysis
Source: Cancer Med. 2021 Nov 19;10(23):8483–96. doi: 10.1002/cam4.4352 (PMC8633220; doi:10.1002/cam4.4352)
Supplement: Supplementary file 3 — Table S1 [file CAM4-10-8483-s001.docx]

Table S1. Univariate and multivariate Competitive risk model analyses for the cancer-special survival in esophageal carcinoma patients before and after inverse probability of treatment weighting.

| Characteristics | Before inverse probability of treatment weighting | | | | After inverse probability of treatment weighting | | | |
| --- | --- | --- | --- | --- | --- | --- | --- | --- |
|  | Univariate analysis | | Multivariate analysis | | Univariate analysis | | Multivariate analysis | |
|  | Hazard ratio  (95% confidence interval) | P-value | Hazard ratio  (95% confidence interval) | P-value | Hazard ratio  (95% confidence interval) | P-value | Hazard ratio  (95% confidence interval) | P-value |
| Surgery |  |  |  |  |  |  |  |  |
| No | Reference |  | Reference |  | Reference |  | Reference |  |
| Yes | 0.64 [0.54, 0.76] | **<0.001** | 0.66 [0.55, 0.79] | **<0.001** | 0.77 [0.71, 0.84] | **<0.001** | 0.82 [0.75, 0.89] | **<0.001** |
| Marital status |  |  |  |  |  |  |  |  |
| Married | Reference |  | Reference |  | Reference |  | Reference |  |
| Others | 1.18 [1.04, 1.33] | **0.010** | 1.14 [1.01, 1.29] | **0.039** | 1.22 [1.12, 1.33] | **<0.001** | 1.25 [1.14, 1.38] | **<0.001** |
| Unknown | 0.85 [0.61, 1.20] | 0.353 | 0.86 [0.61, 1.22] | 0.398 | 0.71 [0.54, 0.95] | **0.019** | 0.77 [0.58, 1.02] | 0.067 |
| Age group |  |  |  |  |  |  |  |  |
| 75-79 years | Reference |  | Reference |  | Reference |  | Reference |  |
| 80-84 years | 1.09 [0.95, 1.24] | 0.206 | 1.09 [0.95, 1.25] | 0.233 | 1.25 [1.14, 1.38] | **<0.001** | 1.25 [1.13, 1.37] | **<0.001** |
| 85+ years | 1.35 [1.12, 1.63] | **0.001** | 1.33 [1.10, 1.61] | **0.004** | 1.17 [1.03, 1.33] | **0.015** | 1.53 [1.32, 1.78] | **<0.001** |
| Race |  |  |  |  |  |  |  |  |
| Black | Reference |  |  |  | Reference |  | Reference |  |
| White | 1.11 [0.76, 1.63] | 0.585 |  |  | 0.78 [0.64, 0.94] | **0.010** | 0.86 [0.71, 1.05] | 0.139 |
| Other | 0.00 [0.00, Inf] | 0.982 |  |  | 0.91 [0.70, 1.19] | 0.500 | 0.84 [0.64, 1.10] | 0.198 |
| Unknown | 1.12 [0.85, 1.49] | 0.410 |  |  | 0.00 [0.00, Inf] | 0.972 | 0.00 [0.00, Inf] | 0.973 |
| Gender |  |  |  |  |  |  |  |  |
| Female | Reference |  |  |  | Reference |  | Reference |  |
| Male | 1.06 [0.93, 1.21] | 0.395 |  |  | 1.22 [1.10, 1.35] | **0.002** | 1.39 [1.24, 1.56] | **<0.001** |
| Year of diagnosis |  |  |  |  |  |  |  |  |
| 2004~2009 | Reference |  | Reference |  | Reference |  | Reference |  |
| 2010~2015 | 0.76 [0.67, 0.85] | **<0.001** | 0.77 [0.69, 0.88] | **<0.001** | 0.78 [0.72, 0.85] | **<0.001** | 0.79 [0.72, 0.86] | **<0.001** |
| Histology |  |  |  |  |  |  |  |  |
| EAC | Reference |  |  |  | Reference |  | Reference |  |
| ESCC | 0.94 [0.83, 1.07] | 0.374 |  |  | 1.15 [1.05, 1.26] | **0.004** | 1.21 [1.09, 1.34] | **0.003** |
| Others | 0.88 [0.69, 1.14] | 0.337 |  |  | 1.02 [0.89, 1.15] | 0.816 | 1.09 [0.93, 1.27] | 0.316 |
| Tumor size |  |  |  |  |  |  |  |  |
| <5cm | Reference |  | Reference |  | Reference |  |  |  |
| >=5cm | 1.20 [1.04, 1.40] | **0.014** | 1.07 [0.92, 1.24] | 0.412 | 1.04 [0.93, 1.15] | 0.503 |  |  |
| Unknown | 1.21 [1.05, 1.40] | **0.009** | 1.12 [0.97, 1.30] | 0.132 | 1.07 [0.97, 1.18] | 0.201 |  |  |
| Grade |  |  |  |  |  |  |  |  |
| I | Reference |  | Reference |  | Reference |  |  |  |
| II | 1.37 [0.94, 2.00] | 0.103 | 1.37 [0.94, 2.00] | 0.105 | 1.30 [0.99, 1.71] | 0.064 |  |  |
| III | 1.58 [1.09, 2.31] | **0.016** | 1.61 [1.10, 2.34] | **0.014** | 1.30 [0.99, 1.72] | 0.061 |  |  |
| IV | 1.21 [0.67, 2.21] | 0.525 | 1.35 [0.74, 2.46] | 0.329 | 1.03 [0.64, 1.65] | 0.912 |  |  |
| Unknown | 1.26 [0.85, 1.86] | 0.251 | 1.23 [0.83, 1.81] | 0.310 | 1.16 [0.87, 1.53] | 0.309 |  |  |
| T stage |  |  |  |  |  |  |  |  |
| T1 | Reference |  | Reference |  | Reference |  | Reference |  |
| T2 | 0.96 [0.74, 1.25] | 0.767 | 1.07 [0.81, 1.40] | 0.649 | 0.93 [0.77, 1.12] | 0.426 | 1.04 [0.85, 1.27] | 0.676 |
| T3 | 1.28 [1.01, 1.64] | **0.044** | 1.46 [1.14, 1.87] | **0.003** | 1.13 [0.95, 1.34] | 0.168 | 1.38 [1.16, 1.66] | **0.004** |
| T4 | 1.97 [1.49, 2.60] | **<0.001** | 2.06 [1.55, 2.74] | **<0.001** | 2.45 [2.01, 2.98] | **<0.001** | 2.70 [2.21, 3.29] | **<0.001** |
| N stage |  |  |  |  |  |  |  |  |
| N0 | Reference |  | Reference |  | Reference |  | Reference |  |
| N1 | 1.12 [0.99, 1.27] | 0.066 | 1.16 [1.02, 1.32] | **0.021** | 1.21 [1.11, 1.32] | **<0.001** | 1.24 [1.13, 1.37] | **<0.001** |
| NX | 3.57 [1.33, 9.55] | **0.011** | 2.25 [0.82, 6.14] | 0.113 | 4.30 [1.61, 11.48] | **0.004** | 2.65 [0.98, 7.16] | 0.055 |

Abbreviations: ESCC, esophageal squamous cell carcinoma; EAC, esophageal adenocarcinoma
